# The association of priori and posteriori dietary patterns with the risk of incident hypertension: Tehran Lipid and Glucose Study

**DOI:** 10.1186/s12967-021-02704-w

**Published:** 2021-01-25

**Authors:** Azra Ramezankhani, Firoozeh Hosseini-Esfahani, Parvin Mirmiran, Fereidoun Azizi, Farzad Hadaegh

**Affiliations:** 1 Prevention of Metabolic Disorders Research Center, Research Institute for Endocrine Sciences, Shahid Beheshti University of Medical Sciences, Tehran, Iran; 2 Nutrition and Endocrine Research Center, Research Institute for Endocrine Sciences, Shahid Beheshti University of Medical Sciences, Tehran, Iran; 3 Endocrine Research Center, Research Institute for Endocrine Sciences, Shahid Beheshti University of Medical Sciences, Tehran, Iran

**Keywords:** Blood pressure, Hypertension, Dietary pattern, Healthy eating index, Factor analysis, Diet

## Abstract

**Background:**

The aim of this study was to investigate the association of dietary patterns with incident hypertension.

**Materials/methods:**

This prospective study was conducted on 4793 individuals of Tehran lipid and glucose study participants, aged ≥ 18 years who were followed for a median of 6.3 years from 2008–2011 to 2016–2018. A valid and reliable semi-quantitative food frequency questionnaire was used to assess usual dietary intakes. Anthropometrics and blood pressure were assessed at baseline and during follow up examinations. Dietary patterns were derived using principal component analysis (PCA). Healthy eating index (HEI) and dietary approach to stop hypertension (DASH) score were measured based on dietary recommendations. Time-dependent Cox models adjusting for confounders were used to examine the association between dietary patterns and the risk of hypertension.

**Results:**

During follow-up, a total of 727 incident cases of hypertension were identified. The mean ± SD age at baseline was 40.3 ± 13.5 and 37.9 ± 12.1 years in men and women, respectively. Two dietary patterns (the healthy and unhealthy) were extracted by PCA. Compared with participants in the first quartile, a 23% (HR: 1.23; 95%CI 1.00–1.53; P trend: 0.056) increased risk of hypertension was found in the fourth quartile of HEI score. This association was disappeared after further adjustment for confounders. Increasing DASH score, the healthy and unhealthy dietary pattern were not associated with risk of hypertension.

**Conclusion:**

Our findings showed that higher adherences to the posteriori- and priori-dietary patterns were not associated with risk of hypertension in this population.

## Background

Hypertension is an important public health concern affecting more than 40% of people aged > 25 years worldwide [1]. It has long been recognized that hypertension is an important risk factor for cardiovascular disease (CVD) and accounts for 50% of all ischemic heart diseases and stroke events (2). Hypertension is a multi-factorial disease resulting from complex interactions between diet, lifestyle and genetic risk factors [2–4]. Most epidemiological studies on the relationships between nutrition and hypertension have focused on a single food/nutrient (e.g. coffee and caffeine, dairy, cruciferous vegetables, etc.) [5, 6]. However, it is often difficult to separate out the specific effects of a single nutrient or food [6, 7]; because, people naturally eat a combination of many different foods that may exhibit complicated interactions or synergistic effects. Therefore, dietary pattern analysis has been suggested as an alternative approach to study the relation between disease and dietary intake [7]. Two methodologies have been suggested: the priori approach in which predefined dietary patterns based on nutrition guidelines or recommendations are used to measure the level of adherence to these patterns. The second approach is based on a posteriori analysis, using multivariate statistical techniques, such as principal component analysis (PCA) and cluster analysis. These methods are completely exploratory which use all available dietary data to extract dietary patterns based on inter-correlations between food groups or nutrients intakes [7, 8].Several investigators in the field of nutrition epidemiology have identified various dietary patterns; e.g. Mediterranean, low salt, low carbohydrate, low fat or dietary approach to stop hypertension diet (DASH), each of them has been shown to have differing effects on human health [9]. The DASH diet was related to the greatest overall decrease in the systolic and diastolic blood pressure (SBP, DBP) [10]. The healthy eating index (HEI) is a diet quality measurement which assesses the conformance of food group intakes to the dietary guidelines. Poor diet quality calculated by HEI has been shown to contribute to the development of hypertension [11]. Moreover, unhealthy dietary patterns identified through PCA were associated with higher BP among Kuwaiti and Lebanese adults [12, 13]. Despite the tremendous increase in the prevalence of hypertension in Iran [14], there are no standard quantitative dietary guidelines to evaluate dietary patterns and their relationships with hypertension. Also, to our knowledge, no study has examined adherence to multiple dietary patterns in a prospective study to determine which pattern have high strength associations with the risk of hypertension among a group of Iranian adults. Also, the amount of variance and interpretability of these patterns as an exposure has not been specified. To address this gap, and to help identifying a feasible and effective dietary pattern to prevent and reduce the risk of hypertension in our country, we prospectively investigated the association between a priori and a posteriori derived dietary patterns and incident hypertension among Tehranian adults participated in the Tehran lipid and glucose study (TLGS).

## Methods

### Study population

Participants for this study were recruited from the TLGS, a large-scale population-based cohort study performed to determine risk factors for non-communicable diseases in a representative sample of residents of Tehran, the capital of Iran. At first phase of the study (1999–2001), 15,005 individuals aged ≥ 3 years were selected using multistage stratified cluster random sampling and follow-up examinations were conducted in five consecutive phases: Phase 2 (2002–2005), Phase 3 (2005–2008), Phase 4 (2008–2011), Phase 5 (2012–2015) and Phase 6 (2015–2018). The details of the study have been published elsewhere [15]. Of 8843 individuals aged ≥ 18 years who participated in Phase 4, a total of 6791 subjects (3016 men) completed the dietary assessment. We selected these subjects as baseline population in this study and followed them at next Phases (Phases 5 and 6). We compared characteristics of adult participants who had dietary data (respondents, n = 6791) and those who did not have (non-respondents, n = 2052). Among respondents, 44.4% were male, 22.6% were current smoker and 5.5% had family history of CVD (FH-CVD), compared with 44.8%, 20.6% and 4.7%, respectively, in non-respondents (P > 0.05). The mean (SD) of age and body mass index (BMI) were 40.8 (14.1) and 27.3 (4.9), respectively, in respondents vs. 44.8 (17.1) and 27.7 (5.2) in non-respondents (P < 0.001). The mean (SD) of SBP and DBP blood pressure among respondents were 114 (16.7) and 75.5 (11.1), respectively, compared with 118 (19.7) and 77.2 (11.5) mmHg in non-respondents (P < 0.001). The level of physical activity did not differ in two groups.

Of 6791 participants, we excluded under- or over-reporters of energy intake (< 800 or ≥ 4200 kcal/day, n = 457), those with hypertension at baseline (n = 1116) and subjects with missing data on hypertension status at baseline (n = 19). Finally, after excluding participants without any follow up data (n = 406), 4793 subjects (1986 men) were remained and entered in the analysis.

### Measurements

At baseline and next phases, information on age, sex, smoking status, medical history and medication use was obtained through a personal interview using a standardized questionnaire [15].

### Anthropometric and blood pressure measurements

Bodyweight was measured using a calibrated digital scale (Seca 707). Height was measured using a portable stadiometer. A qualified physician measured BP using a standard mercury sphygmomanometer after the participants remained seated for 15 min. The calibration of sphygmomanometer was done by Iranian Institute of Standards and Industrial Researches. The cuff was placed on the right arm, which was at the heart level and bloated as high rate as possible increments until the cuff pressure was 30 mmHg, above the level at which the radial pulse disappeared. Two separate measurements were performed with an at least 30-secondinterval time; the mean of two measurements was recorded as the participant's BP. The SBP was determined as the appearance of the first sound [Korotkoff phase 1], and the DBP was determined as the disappearance of the sound [Korotkoff phase 5] during deflating the cuff at a 2–3 mm per second decrement rate [15].

### Physical activity measurements

The physical activity level (PAL) was assessed using the Persian-translated modifiable activity questionnaire (MAQ) with high reliability and relative validity [16].

### Dietary intake assessment

Dietary data were collected at baseline through face-to-face personal interviews with the use of a valid and reliable 168-items semi-quantitative food frequency questionnaire (FFQ). Participants used the standard serving sizes to report the usual frequency of consumption of individual food items on a daily, weekly, or monthly basis during the last year. The usual food intakes were then converted to daily intake (in grams) and were calculated in energy-adjusted terms (serving per 1000 kcal/day). Because the Iranian food composition table (FCT) is incomplete, the United States Department of Agriculture (USDA) FCT was used to analysis of food composition [17]. Foods listed in the FFQ were collapsed into 20 mutually exclusive food categories based on the similarity of type of food and nutrient composition.

### DASH score

The DASH diet is a dietary pattern originally developed to prevent and control hypertension. DASH score is an index for measuring diet quality [18]. We computed a 40-points DASH score which includes 8 dietary components [19]. All components were computed per 1000 kcal and were then divided into quintiles. Each quintile intake received 1 point. For fruits, vegetables, whole grains, low-fat dairy, nuts and legumes, a score 5 was given to those in the top quintile. For sodium, red and processed meats, and sweetened beverages, the lowest quintile was given a score of 5, and the top quintile was given a score of one. The overall DASH score was then obtained by adding the component scores ranging from 8 to 40. A higher DASH score indicates better adherence.

### HEI score

HEI is based on key recommendations of the 2015-2020 dietary guidelines for Americans (DGA). It is comprised of 13 dietary components. Nine adequacy components include total fruits, whole fruits, total vegetables, greens and beans, whole grains, dairy, total protein foods, seafood and plant proteins, and fatty acids. Four moderation components (those that should be limited) include refined grains, sodium, added sugars, and saturated fats [20]. The HEI scoring is based on density (amount per 1000 kcal, ratio of fatty acids) and recommendations are in the range of 1200–2400 kcal dietary intake. To compute the score of HEI, six components from nine adequacy components (total fruit, whole fruit, total vegetables, greens and beans, total protein foods and seafood and plant proteins) each received a score of 0 and 5 respectively for the lowest and highest consumption. The other three adequacy components (whole grains, dairy and fatty acids) were scored from 0 to 10 for the lowest and highest consumption, respectively.

An individual who had an amount of zero per 1000 kcal receive a minimum score of zero; therefore, densities of exactly half the standard for maximum points would interpret to a score of half the possible points (e.g., 5 out of 10 score).

The four moderation components (refined grains, sodium, added sugars, and saturated fats) received a score of 10 and 0 for the lowest and highest intakes, respectively. Intermediate intakes between the minimum and maximum were prorated. The scores from the 13 components were added for a total HEI score ranging from 0 to 100. Higher total HEI scores indicate greater adherence to DGA recommendations [20].

### Laboratory assays

Blood samples were collected after a 12- to 14-h overnight fasting. Fasting plasma glucose (FPG) was measured using an enzymatic colorimetric method with glucose oxidase. Triglyceride (TG) concentrations were assayed with glycerol phosphate oxidase using the enzymatic colorimetric method. Analyses were performed using Pars Azmoon kits (Pars Azmoon Inc., Tehran, Iran) and a Selectra 2 auto-analyser (Vital Scientific, Spankeren, theNetherlands). Inter- and intra-assay coefficients of variation (CVs) were both < 2.2% for FPG, 0.6% and 1.6% for TG, respectively [15].

### Definition of terms and outcome

Smoking status was categorized as smoker (current smokers) versus non-smoker (including past and never-smokers). A current smoker was defined as a person who smokes cigarettes or other smoking implements daily or occasionally. A positive FH-CVD was defined as diagnosis of CVD in a male first degree relative < 55 or in a female first degree relative < 65 years. Individuals were considered physically active when they achieved a minimum of at least 600 MET (metabolic equivalent task)- minutes per week [21]. Type 2 diabetes mellitus (DM) was defined as FPG ≥ 7 mmol/L or 2 h-PLPG ≥ 11.1 mmol/L [22] or using glucose-lowering treatment. Hypertension was defined as a SBP ≥140 mmHg or a DBP ≥90 mmHg or taking antihypertensive medications [23].

### Statistical methods

Missing data among total population (after applying the exclusion criteria) were 1.1, 1.1, 0.1, 2.4 and %9.6 for baseline covariates including smoking status, BMI, TC, DM status and PAL, respectively. Thus, multivariate imputations by chained equations (MICE) (mice package in R software) [24] were used to impute missing values at baseline. The PCA was used as a posteriori method with orthogonal rotation to identify dietary patterns on 20 food groups (as servings per 1000 kcal/day). Eigenvalues > 1 derived from the correlation matrix, scree plots, factor interpretability and variance explained > 5% were used to extract key dietary patterns. Food groups with absolute factor loadings values > 0.2 were considered as contributing highly to the extracted pattern. Each person received a factor score for each dietary pattern by summing intakes of food groups weighted by the loadings generated by the PCA. The posteriori and priori dietary patterns (DASH and HEI) scores were then stratified into quartiles. The baseline characteristics of the study population were compared across quartile categories of each dietary pattern using descriptive analysis. To test linear trend for categorical and continuous variables across quartiles of dietary pattern scores, logistic and linear regression tests were performed respectively; with the use of quartiles of dietary pattern scores as a continuous variable in those models. The incidence density rate of hypertension was calculated by dividing the number of events by the person-years at risk.

The association between different dietary patterns and incidence of hypertension was analyzed using time-dependent Cox proportional hazard (Cox PH) regression. The interaction of dietary patterns and age/sex in relation to hypertension were not significant. All covariates (excluding sex and dietary patterns) were included in the models as time-dependent variables. Missing data on time-dependent variables was imputed by the last observation carried forward (LOCF) approach. For these analyses, the lowest quartiles of the different dietary patterns were considered as the reference category. Time to event was defined as the time between baseline and the event date (for event cases) or the last follow-up (for censored cases), whichever occurred first. The event date was defined as the mid-time between the date of the follow-up visit at which hypertension was detected for the first time, and the most recent follow-up visit prior to the diagnosis. Study participants were censored due to death, loss to follow-up or non-occurrence of hypertension before the end of the follow-up (18th April 2018). Two models were developed; model 1 was adjusted for the age and sex. Model 2 was further adjusted for time dependent BMI, smoking, DM status, PAL, TG, FH-CVD, total energy and salt intakes as the most important confounders. The PH assumption was verified using Schoenfeld residuals test and plot of log [− log (survival)] versus log (time) to see if they are parallel. We conducted tests for linear trends with the use of quartiles of dietary patterns as a continuous variable and modeled this variable in separate Cox PH models. Analyses were conducted with R software (version 3.6.2) and the Statistical Package for Social Sciences (SPSS; version 21.0), and a two sided P values < 0.05 were considered statistically significant.

## Results

Of 4793 participants, 58.6% were women, and the mean (SD) age at baseline was 40.3 (13.5) and 37.9 (12.1) years in men and women, respectively. During a median follow-up of 6.3 years (inter-quartile range: 5.7–6.9), 727 incident cases of hypertension (343 in men and 384 in women) occurred. Incidence rate (95% confidence interval (CI)) was 27.1 (25.2–29.2), 31.4 (28.2–34.9), and 24.2 (21.9–26.7) per 1000 person years in total population, men and women, respectively.

Two dietary patterns were extracted by PCA; a healthy dietary pattern which was positively associated with the consumption of nuts and seeds, low fat dairy products, vegetables, fruits, poultry and fish; and an unhealthy dietary pattern characterized by foods such as red meat, fast food, high fat dairy products, refined grain, salty snacks, and drinks (Table 1).

**Table 1:** Factor loadings for two dietary patterns identified by principal component analysis among the participants of the Tehran Lipid and Glucose Study

Food groups	Healthy	Unhealthy
Tea and coffee		0.24
Sugar sweetened beverages	− 0.20	0.68
Legumes		− 0.22
Red meats		0.28
Nuts and seeds	0.24	0.20
Fast foods		0.51
Low fat dairy products	0.26	− 0.29
High fat dairy products		0.21
Green vegetables	0.61	
Yellow and red vegetables	0.69	
Other vegetables	0.72	
Total fruit	0.43	
Fruit juices	0.45	− 0.24
Refined grains	− 0.61	0.29
Whole grains		− 0.30
Salty snacks		0.20
Poultry and fish	0.24	
Added sugars		0.69
Liquid oils	0.37	0.22
Solid oils	− 0.25	
% variance	12.7	9.84

The mean score of HEI and DASH were 63.1 ± 8.92 and 23.0 ± 3.80, respectively. Baseline characteristics of participants across quartiles of each dietary pattern scores are shown in Table 2. Participants in higher quartiles of all dietary patterns except the unhealthy dietary pattern were older, compared to those in the lowest quartile. Also, higher quartiles of the healthy dietary pattern and HEI scores were associated with lower BMI. In addition, higher quartiles of all dietary patterns, except the unhealthy dietary pattern were associated with higher levels of physical activity and lower proportion of current smoker. However, higher quartiles of the healthy dietary pattern and DASH scores were associated with higher proportion of diabetes. Baseline characteristic of participants who developed and did not develop hypertension is shown in Additional file 1: Table S1 by sex. Men and women who developed hypertension had higher mean of age, BMI, TG, SBP and DBP than subjects who did not develop hypertension. Also the prevalence of diabetes and current smoking were higher among those individuals who developed hypertension.

**Table 2:** Baseline characteristics of participants by quartiles of posteriori and priori dietary patterns in the participants of the Tehran Lipid and Glucose Study (n = 4793)

	Quartiles of healthy dietary pattern score				P trend	Quartiles of unhealthy dietary pattern score				P trend
	1	2	3	4		1	2	3	4	
n	1198	1198	1199	1198		1198	1198	1199	1198	
Age (years)	36.5 (12.5)	37.6 (12.8)	39.4 (12.6)	42.1 (12.5)	< 0.001	43.4 (13.1)	40.1 (12.8)	37.0 (12.0)	35.1 (11.7)	< 0.001
BMI (kg/m^2^)	27.7 (4.7)	26.7 (4.2)	26.2 (4.4)	26.2 (4.4)	< 0.001	26.1 (4.6)	26.9 (4.5)	26.7 (4.7)	26.0(4.4)	0.810
TG (mmol/L)	1.51 (0.93)	1.47 (1.45)	1.46 (0.88)	1.53 (0.94)	0.66	1.43 (0.8)	1.45 (1.0)	1.54 (1.4)	1.56 (0.91)	0.017
SBP (mmHg)	108 (11.6)	108 (11.8)	108 (12.2)	108 (11.8)	0.713	104 (12.5)	109(11.8)	108 (11.5)	107 (11.5)	0.001
DBP (mmHg)	75.0 (8.2)	73.12 (8.7)	73.18 (8.3)	72.5 (8.45)	0.310	73.1 (8.4)	73.1 (8.6)	72.8 (8.4)	72.7 (8.4)	0.412
Low PAL (%)	75.6	73.2	70.8	64.9	< 0.001	69.9	70.8	71.6	72.3	0.621
FH-CVD (%)	4.8	4.4	5.6	6.3	0.170	4.8	5.2	5.4	5.6	0.854
Diabetes (%)	4.8	3.9	5.0	7.9	< 0.001	9.1	5.8	4.2	2.5	< 0.001
Current smoker (%)	30	25.4	19.8	13.9	< 0.001	14.6	17.0	24.4	35.1	< 0.001

	Quartile of DASH score				P trend	Quartile of HEI-2015 score				P trend
	1	2	3	4		1	2	3	4	
n	1254	932	1328	1279		1212	1231	1183	1167	
Age (years)	34.1 (11.46)	37.5 (11.9)	39.6 (12.5)	44.1 (12.9)	< 0.001	36.9 (12.1)	37.7 (12.4)	39.3 (12.8)	41.9 (13.2)	< 0.001
BMI (kg/m^2^)	26.0 (4.6)	26.4 (4.5)	26.7 (4.4)	27.5 (4.51)	< 0.001	27.4 (4.5)	26.5 (4.5)	26.4 (4.5)	26.3 (4.6)	< 0.001
TG (mmol/L)	1.40 (0.8)	1.49 (0.9)	1.52 (1.4)	1.53 (0.93)	0.007	1.40 (0.85)	1.51 (1.47)	1.49 (0.89)	1.52 (1.01)	0.84
SBP (mmHg)	109 (12.0)	108 (11.6)	109 (12.0)	107 (11.7)	< 0.001	109 (11.8)	108 (11.9)	108 (12.0)	107 (11.7)	< 0.001
DBP (mmHg)	72.5 (8.5)	72.9 (8.64)	73.1 (8.52)	73.2 (8.2)	0.742	73.01 (8.5)	72.6 (8.5)	72.8 (8.5)	73.3 (8.2)	0.120
Low PAL (%)	75.2	74.5	71.5	64.3	< 0.001	76.3	72.9	68.8	66.3	< 0.001
FH-CVD (%)	6.2	5.8	5.6	5.3	0.612	6.2	5.3	4.9	4.6	0.342
Diabetes (%)	3.3	5.0	5.4	7.7	< 0.001	3.7	4.8	4.4	3.9	0. 654
Current smoker (%)	32.5	25.8	19.3	13.4	< 0.001	26.6	24.0	22.1	16.2	< 0.001

Baseline characteristic of male and female participants by quartiles of priori- and posteriori dietary patterns are shown in Additional file 1: Table S2 and S3.

Baseline dietary intakes of participants who developed and did not develop hypertension are presented in Additional file 1: Table S4. Participants who developed hypertension had higher intakes of added sugar, red meats and low fat dairy intake. Subjects who did not develop hypertension had higher intake of legumes, fast food, carbonated drinks and energy intake.

Dietary intakes of participants across quartiles of all dietary pattern scores are presented in Tables 3 and 4. Participants in the top quartiles of the healthy dietary pattern, DASH and HEI scores had higher intakes of liquid oil, legumes, nuts and seeds, low fat dairy products, vegetables and total fruit, compared to those in the lowest; however, intakes of solid oil, added sugars, fast food, refined grains, and sweetened drinks tend to decrease across quartiles of these dietary pattern scores. In addition, individuals with higher scores of the unhealthy dietary pattern had higher intakes of solid oil, added sugars, red meats, fast food, high fat dairy products, salty snacks, sweetened drinks, and energy intake.

**Table 3:** Dietary intakes of participants by quartiles of posteriori dietary patterns in the participants of the Tehran Lipid and Glucose Study (n = 4793)

	Quartiles of healthy dietary pattern score				P trend	Quartiles of unhealthy dietary pattern score				P trend
	1	2	3	4		1	2	3	4	
n	1198	1198	1199	1198		1198	1198	1199	1198	
Dietary intakes										
Liquid oils (gr/1000 kcal)	2.83 (2.0)	3.68 (2.4)	4.35 (2.8)	5.54 (4.1)	< 0.001	3.24 (2.2)	3.90 (2.5)	4.33 (3.0)	4.93 (4.1)	0.104
Solid oils (gr/1000 kcal)	7.91 (7.1)	6.26 (5.6)	5.29 (5.2)	3.88 (4.7)	< 0.001	4.82 (5.6)	5.97 (6.0)	6.28 (6.1)	6.27 (5.8)	< 0.001
Added sugars (% of energy)	5.39 (3.4)	5.45 (3.3)	5.07 (2.9)	4.24 (2.6)	< 0.001	2.71 (1.4)	4.01 (1.8)	5.36 (2.2)	8.07 (3.6)	< 0.001
Legumes^a^	0.15 (0.1)	0.18 (0.1)	0.20 (0.1)	0.22 (0.2)	< 0.001	0.24 (0.2)	0.19 (0.1)	0.18 (0.1)	0.15 (0.1)	< 0.001
Red meats^a^	0.32 (0.2)	0.37 (0.2)	0.41 (0.3)	0.41 (0.3)	< 0.001	0.28 (0.1)	0.35 (0.2)	0.41 (0.2)	0.48 (0.3)	< 0.001
Nuts and seeds^a^	0.13 (0.2)	0.18 (0.2)	0.22 (0.3)	0.29 (0.4)	< 0.001	0.14 (0.1)	0.20 (0.2)	0.22 (0.3)	0.28 (0.4)	< 0.001
Fast foods^a^	0.16 (0.2)	0.18 (0.2)	0.17 (0.1)	0.14 (0.1)	0.004	0.07 (0.07)	0.12 (0.0)	0.17 (0.1)	0.28 (0.2)	< 0.001
Low fat dairy products^a^	0.49 (0.3)	0.58 (0.4)	0.62 (0.3)	0.73 (0.4)	< 0.001	0.77 (0.4)	0.62 (0.3)	0.57 (0.3)	0.46 (0.3)	< 0.001
High fat dairy products^a^	0.31 (0.3)	0.34 (0.3)	0.35 (0.3)	0.32 (0.3)	0.39	0.21 (0.2)	0.33 (0.3)	0.37 (0.3)	0.39 (0.3)	< 0.001
Green vegetables^a^	0.14 (0.1)	0.21 (0.1)	0.29 (0.2)	0.43 (0.3)	< 0.001	0.29 (0.2)	0.29 (0.2)	0.27 (0.2)	0.23 (0.1)	< 0.001
Yellow and red vegetables^a^	0.15 (0.1)	0.24 (0.1)	0.32 (0.2)	0.53 (0.3)	< 0.001	0.36 (0.3)	0.32 (0.2)	0.30 (0.2)	0.25 (0.2)	< 0.001
Other vegetables^a^	0.38 (0.1)	0.55 (0.2)	0.74 (0.3)	1.08 (0.5)	< 0.001	0.75 (0.5)	0.72 (0.4)	0.69 (0.4)	0.60 (0.4)	< 0.001
Refined grains^a^	5.62 (1.6)	4.19 (1.3)	3.56 (1.2)	2.74 (1.2)	< 0.001	4.68 (2.03)	4.17 (1.6)	3.76 (1.5)	3.49 (1.3)	< 0.001
Whole grains^a^	0.75 (1.0)	0.83 (1.1)	0.78 (0.98)	0.78 (0.9)	0.42	1.28 (1.4)	0.78 (0.9)	0.59 (0.69)	0.49 (0.5)	< 0.001
Salty snacks^a^	0.11 (0.1)	0.13 (0.1)	0.14 (0.3)	0.13 (0.2)	< 0.001	0.08 (0.1)	0.11 (0.1)	0.14 (0.1)	0.18 (0.4)	< 0.001
Poultry and fish^a^	0.45 (0.3)	0.56 (0.4)	0.64 (0.5)	0.76 (0.7)	< 0.001	0.53 (0.4)	0.59 (0.5)	0.64 (0.6)	0.65 (0.6)	0. 276
Tea and coffee (gr/day)	559 (461)	577 (434)	599 (434)	599 (463)	0.006	443 (323)	523 (366)	606 (444)	733 (586)	< 0.001
Carbonated drinks (gr/day)	62.5 (85.9)	53.8 (70.1)	42.6 (59.0)	29.6 (46.2)	< 0.001	12.3 (14.7)	22.4 (24.9)	40.9 (41.2)	113 (99.5)	< 0.001
Total fruit^a^	0.70 (0.5)	1.07 (0.7)	1.30 (0.8)	1.62 (0.9)	< 0.001	1.10 (0.8)	1.23 (0.8)	1.23 (0.9)	1.11 (0.8)	0.73
Fruits juice*	0.07 (0.1)	0.11 (0.2)	0.13 (0.2)	0.15 (0.2)	< 0.001	0.08 (0.1)	0.11 (0.16)	0.12 (0.18)	0.14 (0.19)	< 0.001
Salt (gr/1000 kcal)	2.46 (2.0)	2.91 (2.0)	2.71 (2.1)	2.71 (2.0)	0.18	2.39 (1.8)	2.53 (1.8)	2.96 (1.9)	2.90 (2.3)	< 0.001
Total energy (kcal/day)	2473 (722)	2445 (712)	2378 (694)	2295 (718)	< 0.001	2282 (698)	2323 (709)	2416 (706)	2570 (713)	< 0.001

P trend was calculated by treating quartiles of food groups and energy intake as a continuous variable in the linear regression model. Values are reported as mean (SD)

^a^Servings per 1000 kcal

**Table 4:** Dietary intakes of participants by quartiles of a priori dietary patterns in the participants of the Tehran Lipid and Glucose Study (n = 4793)

	Quartiles of DASH score					Quartiles of HEI score				
	1	2	3	4	P trend	1	2	3	4	P trend
n	1254	932	1328	1279		1212	1231	1183	1167	
Mean score	18.2 ± 1.72	21.5 ± 0.50	24.0 ± 0.81	27.7 ± 1.75		51.9 ± 4.49	60.2 ± 1.67	66.0 ± 1.84	74.7 ± 4.21	
Dietary intakes										
Liquid oils (gr/1000 kcal)	4.09 (3.0)	4.04 (2.9)	4.11 (3.7)	4.17 (2.6)	0.003	3.69 (2.8)	3.98 (3.0)	4.22 (2.8)	4.54 (3.7)	0.001
Solid oils (gr/1000 kcal)	7.05 (6.4)	6.40 (6.1)	5.77 (5.3)	4.29 (4.3)	< 0.001	7.32 (6.9)	5.88 (5.6)	5.39 (5.3)	4.70 (5.2)	< 0.001
Added sugars (% of energy)	6.15 (3.4)	5.29 (3.0)	4.80 (3.3)	4.01 (2.8)	< 0.001	5.85 (3.7)	5.23 (3.2)	4.77 (2.7)	4.26 (2.4)	< 0.001
Legumes^a^	0.14 (0.1)	0.17 (0.1)	0.19 (0.1)	0.25 (0.2)	< 0.001	0.14 (0.1)	0.18 (0.1)	0.20 (0.1)	0.25 (0.2)	< 0.001
Red meats^a^	0.45 (0.3)	0.41 (0.2)	0.37 (0.2)	0.31 (0.3)	< 0.001	0.37 (0.2)	0.39 (0.3)	0.38 (0.2)	0.38 (0.3)	0.10
Nuts and seeds^a^	0.15 (0.2)	0.19 (0.3)	0.21 (0.2)	0.28 (0.3)	< 0.001	0.16 (0.2)	0.20 (0.3)	0.21(0.2)	0.26 (0.3)	< 0.001
Fast foods^a^	0.24 (0.2)	0.17 (0.1)	0.14 (0.2)	0.09 (0.1)	< 0.001	0.18 (0.2)	0.17 (0.2)	0.16 (0.2)	0.13 (0.1)	< 0.001
Low fat dairy products^a^	0.44 (0.3)	0.55 (0.3)	0.64 (0.3)	0.77 (0.3)	< 0.001	0.56 (0.4)	0.59 (0.4)	0.61 (0.4)	0.67 (0.4)	< 0.001
High fat dairyproducts*	0.35 (0.3)	0.32 (0.3)	0.32 (0.2)	0.30 (0.3)	0.010	0.36 (0.3)	0.33 (0.3)	0.31 (0.3)	0.30 (0.3)	< 0.001
Green vegetables^a^	0.20 (0.2)	0.24 (0.1)	0.28 (0.2)	0.35 (0.2)	< 0.001	0.21(0.2)	0.26 (0.2)	0.28 (0.2)	0.34 (0.2)	< 0.001
Yellow and red vegetables*	0.21 (0.2)	0.26 (0.2)	0.32 (0.2)	0.42 (0.2)	< 0.001	0.24 (0.2)	0.29 (0.2)	0.32 (0.2)	0.38 (0.3)	< 0.001
Other vegetables^a^	0.52 (0.3)	0.62 (0.3)	0.70 (0.3)	0.89 (0.4)	< 0.001	0.57 (0.4)	0.67 (0.4)	0.70 (0.4)	0.82 (0.5)	< 0.001
Refined grains^a^	4.72 (1.7)	4.36 (1.7)	3.94 (1.6)	4.02 (1.4)	< 0.001	5.13 (1.6)	4.49 (1.6)	3.79 (1.4)	2.62 (1.1)	< 0.001
Whole grains^a^	0.43 (0.7)	0.71 (1.0)	0.89 (1.2)	1.09 (1.1)	< 0.001	0.32 (0.5)	0.54 (0.7)	0.89 (1.05)	1.44 (1.3)	< 0.001
Salty snacks^a^	0.16 (0.2)	0.13 (0.1)	0.12 (0.4)	0.10 (0.1)	< 0.001	0.14 (0.3)	0.13 (0.1)	0.12 (0.12)	0.11 (0.3)	< 0.001
Poultry and fish^a^	0.62 (0.6)	0.62 (0.6)	0.58 (0.5)	0.60 (0.6)	0.21	0.49 (0.4)	0.54 (0.4)	0.64 (0.57)	0.75 (0.6)	< 0.001
Tea and coffee(gr/day)	568 (460)	583 (475)	581 (447)	576 (441)	0.33	571 (466)	600 (471)	567 (428)	567 (449)	0.91
Carbonated drinks (gr/day)	85.6 (90.7)	50.2 (62.0)	36.7 (55.2)	18.1 (31.7)	< 0.001	59.2 (83.9)	49.9 (70.0)	42.8 (58.4)	36.0 (53.8)	< 0.001
Total fruit^a^	0.74 (0.6)	0.72 (0.23)	0.80 (0.22)	0.87 (0.24)	< 0.001	0.74 (0.6)	1.07 (0.7)	1.27 (0.8)	1.62 (0.9)	< 0.001
Fruits juice^a^	0.09 (0.14)	0.11 (0.17)	0.12 (0.17)	0.14 (0.20)	< 0.001	0.09 (0.1)	0.11 (0.15)	0.13 (0.2)	0.15 (0.2)	< 0.001
Salt (gr/1000 kcal)	4.48 (1.5)	4.37 (1.7)	2.52 (1.8)	2.39 (1.8)	< 0.001	3.45 (7.4)	2.76 (2.10)	2.48 (1.8)	2.07 (1.7)	< 0.001
Total energy (kcal/day)	2411 (708)	2376 (736)	2400 (713)	2399 (700)	0.48	2315 (690)	2382 (734)	2427 (715)	2471 (711)	< 0.001

*DASH* Dietary Approaches to Stop Hypertension, *HEI* healthy eating index; P trend was calculated by treating quartiles of dietary patterns scores as a continuous variable in the linear regression model. Values are reported as mean (SD)

^a^Servings per 1000 kcal

Dietary intakes of participants by quartiles of priori- and posteriori dietary patterns among male participants are shown in Additional file 1: Tables S5 and S6.

Dietary intakes of participants by quartiles of extracted dietary patterns by PCA and priori dietary patterns among female participants are shown in Additional file 1: Tables S7 and S8.

The multivariable-adjusted HRs for the risk of hypertension across quartiles of dietary pattern scores are shown in Table 5.

**Table 5:** Hazard ratios of hypertension in relation to quartiles of different dietary patterns in the participants of the Tehran Lipid and Glucose Study (n = 4793)

	Quartiles of dietary patterns				P trend
	Q1	Q2	Q3	Q4	
Healthy					
Incident hypertension/ Number at baseline	160/1198	166/1198	189/1199	212/1198	
Model 1	Reference	0.94(0.76–1.17)	1.02(0.82–1.26)	1.13(0.91–1.39)	0.166
Model 2	Reference	0.93(0.75–1.16)	0.97 (0.77–1.21)	1.15 (0.90–1.47)	0.231
Unhealthy					
Incident hypertension/ Number at baseline	226/1198	204/1198	154/1199	143/1198	
Model 1	Reference	1.10 (0.91–1.34)	0.92 (0.74–1.14)	1.04 (0.83–1.30)	0.854
Model 2	Reference	1.11 (0.91–1.34)	0.94 (0.76–1.18)	1.01 (0.77–1.33)	0.795
DASH					
Incident hypertension/ Number at baseline	129/1254	135/932	211/1328	252/1279	
Model 1	Reference	1.20 (0.94–1.54)	1.18(0.94–1.48)	1.19 (0.96–1.49)	0.168
Model 2	Reference	1.18 (0.93–1.51)	1.12 (0.89–1.41)	1.19 (0.95–1.49)	0.197
HEI					
Incident hypertension/ Number at baseline	149/1212	173/1231	184/1183	221/1167	
Model 1	Reference	1.10 (0.88–1.38)	1.12 (0.90–1.40)	1.23 (1.00–1.53)*	0.056
Model 2	Reference	1.07 (0.86–1.33)	1.13 (0.91–1.41)	1.19 (0.96–1.48)	0.082

Model 1: Adjusted for age and sex

Model 2: Adjusted for age, sex, and time dependent body mass index, smoking status, diabetes status, physical activity, triglycerides, family history of cardiovascular disease, salt and total energy intake

For the test for trend, we treated quartiles of dietary patterns scores as a continuous variable and modeled this variable in the Cox regression analyses

*DASH* Dietary Approaches to Stop Hypertension, *HEI* healthy eating index

Participants in the highest quartile of HEI score had a 23% (HR: 1.23; 95% CI: 1.00–1.53; P trend: 0.056) increased risk of hypertension, compared with individuals in the lowest quartile. However, the association was attenuated (1.19; 0.96–1.48) and became non-significant after further adjustment for confounders (P trend: 0.082). Increasing DASH score and the healthy dietary pattern were not associated with reduced risk of hypertension. Further, no significant association was observed between the unhealthy dietary pattern and incidence of hypertension.

## Discussion

According to the results of this large prospective cohort study, adherence to the posteriori-derived dietary patterns (healthy and unhealthy dietary pattern) and the priori dietary patterns (HEI and DASH) was not associated with the risk of hypertension after adjustment for confounding factors. Participants in the highest quartile of HEI score had a 23% increased risk of hypertension compared to individuals in the lowest quartile; however, this association became non-significant after further adjustment for confounding factors.

Generally, we did not find a significant relationship between the derived healthy dietary pattern and risk of hypertension. Although the posteriori dietary patterns in our study cannot be compared directly with those of other studies, anon-significant relationship between the healthy dietary pattern and risk of hypertension has also been reported in several studies [5, 25], which was consistent with our findings. However, the pooled results from a meta-analysis indicated that empirically derived healthy dietary pattern was associated with a decreased odds of hypertension [3]. Lack of association between the healthy dietary pattern and hypertension in our study may be due to the interactions among different food groups. For example, in our study higher adherence to the healthy dietary pattern were characterized by higher intakes of healthy food groups and higher consumption of some unhealthy food groups such as salty snacks and red meats, which may nullify the benefits of the healthy food groups. Moreover, whole grains with anti-inflammatory and antioxidant effects which could reduce vascular oxidative stress, endothelial impairment, arterial stiffness, and lower BP [26, 27], was not associated with higher healthy dietary pattern score (Table 3). Previous studies have shown that each additional daily 100 g intake of red meat was associated with 14% increased risk of hypertension. This may be due to Millard reaction products in cooked meat through its inflammatory and oxidation pathways [28]. Also, evidence from observational studies has reported strong positive association between daily sodium intake and BP [29]. Furthermore, lack of association may be due to the cooking methods or added fats which could contribute toward the neutralizing the beneficial effects of healthy foods [30]. Finally, participants in the higher quartiles of healthy dietary pattern scores were older and had higher prevalence of diabetes than their counterparts in the lowest quartiles; which are two important factors for increasing hypertension; a finding that was also observed in Asghari et al. study [31].

In our study, the overall risk increment observed with higher HEI score in the crude model which attenuated after adjustment for energy intake. This unexpected result may be due to this fact that HEI assess diet quality independent of diet quantity; it evaluates density rather than absolute amounts, so the interpretation of HEI score depends on whether a person has achieved energy balance. Also, HEI score evaluates merged of foods which collectively reveal a pattern with a single aspect of diet quality; indeed the patterns of diet quality can be different in each person and additional information enclosed in the pattern rather than overall score should be pointed [32, 33].

The unhealthy dietary pattern was not associated with the incidence of hypertension in our study which was inconsistent with Monge A. et al. study in Mexican women [34] and Park JE et al. study in Korean adults [35]; however our results were consistent with previous meta-analysis study in which western-style or unhealthy dietary pattern was not related to the risk of hypertension [3]. This may be due to some food groups like high-fat dairy products and nuts which contain calcium, potassium and other vitamins in combination with unhealthy foods that counteract with the harmful effect of unhealthy style pattern and change this relationship to non-significant [3].

Based on our results, DASH eating pattern, a diet rich in fruit and vegetable, had no relationship with incidence of hypertension. This finding contrasts with the findings of previous clinical trial studies that demonstrated the influence of DASH diet on decreasing SBP [36, 37]. In two randomized clinical trials (RCT), the DASH diet had beneficial effects on decreasing BP among diabetic patients [37] and individuals with the metabolic syndrome [38]. Four points can be noted in our findings to justify this difference. First, we computed DASH score by scoring quintiles of intakes of the eight main components; it is possible that the average of each food group intakes in the top quintile of our study was inconsistent with other studies. Also based on rankings of dietary intake score, individuals who are in the fourth quartile intake for each food group might not meet the current recommendations of that food group. Second, meta-analysis of RCT studies reported that DASH diet can be more effective in hypertensive persons than in normotensive people [29, 36]; and third, the beneficial effect of a DASH diet on BP depends on its ability to restrict calorie intake and weight loss [29, 36]; however, in our study, energy intake increased with higher adherence to DASH diet. Fourth, the applied DASH eating pattern was not homogeneous in different studies, and the characteristic of DASH diet in our study may differ from other studies due to diversity of food groups eaten in different countries [39]. Considering the above mentioned issues, it may be more effective to score dietary patterns based on recommended amount of local food groups to reduce the population risk effectively [39].

In our study, none of the overall eating patterns obtained by the posteriori or priori approach had significant association with increased risk of hypertension, suggesting the need to adapt or develop a score that tailors to local diets. On the other hand, hypertension is a complex concept and has multidimensional aspects being affected by multiple unknown or unmeasured lifestyle factors that are not accounted for analyzing the relationship of dietary patterns and hypertension in our study. Furthermore, we did not include genetic factors which have an important role in the development of hypertension [40]. It is suggested to consider the interaction of various genetic markers and lifestyle factors such as diet in determining hypertension in future studies. Finally, categorizing study samples by quartiles of dietary pattern scores may affect statistical power in the HR context; as the statistical power of our study to detect a HR of 20 and 30% were about 41 and 71% in last quartile of all dietary pattern scores.

To our knowledge, this is the first prospective study to examine the relation of two kinds of dietary patterns (a priori and a posterior dietary pattern) and incidence of hypertension in the Middle East. Strengths of our study include cohort design and detailed information on physical activity, BMI, smoking status, diabetes status and lipid profile, which allowed extensive adjustment for hypertension risk factors. Certain limitations of this study need to be considered. First, the small number of incident events precluded stratification of analyses by sex. Second, this study was performed on residents of district 13 of Tehran that may not necessarily be representative of the general Iranian populations. Third, as inherent to any dietary pattern analysis study, we assessed dietary patterns at baseline; however, dietary patterns may change over time, leading to biased estimated hazard ratios towards the null. Fourth, statistical methods used to define posteriori-dietary pattern, i.e., PCA, are somewhat subjective and dietary patterns can vary by socioeconomic status, ethnic groups and cultures. Also, we did not have enough information about family income; therefore, in our study, it was not possible to investigate the effect of family income on dietary patterns. For future studies, it is recommended to expand this research with larger sample sizes and a wide range of confounders in other populations of Iranian adults.

## Conclusions

Our study showed no association between priori and posteriori-derived dietary patterns and risk of incident hypertension in this population. These results are of considerable public health importance for promoting local dietary guidelines and new index scores to monitor the quality of food consumption in individuals and population groups, especially in our country with high prevalence of hypertension.

## Supplementary Information

**Additional file 1: Table S1.** Baseline characteristics of participants who developed and did not develop hypertension in the participants of the Tehran Lipid and Glucose Study (n=4793). **Table S2**. Baseline characteristics of male participants by quartiles of different dietary patterns in the Tehran Lipid and Glucose Study (n=1986).** Table S3.** Baseline characteristics of female participants by quartiles of different dietary patterns in the Tehran Lipid and Glucose Study (n=2807).** Table S4.** Baseline dietary intake of participants who developed and did not develop hypertension in the Tehran Lipid and Glucose Study (n=4793).** Table S5.** Dietary intakes of participants by quartiles of extracted dietary patterns by PCA among male participants of the Tehran Lipid and Glucose Study (n=1986).** Table S6.** Dietary intakes of participants by quartiles of a priori dietary patterns among male participants of the Tehran Lipid and Glucose Study (n=1986). ** Table S7.** Dietary intakes of participants by quartiles of extracted dietary patterns by PCA among female participants of the Tehran Lipid and Glucose Study (n=2807). ** Table S8.** Dietary intakes of participants by quartiles of a priori dietary patterns among female participants of the Tehran Lipid and Glucose Study (n=2807)..

## Data Availability

The datasets used and/or analyzed during the current study are available from the corresponding author on reasonable request.

## Abbreviations

2-h PLPG: 2-H post load plasma glucose
BMI: Body mass index
CVD: Cardiovascular disease
DASH: Dietary approach to stop hypertension
DBP: Diastolic blood pressure
DGA: Dietary guidelines for Americans
DM: Diabetes mellitus
FCT: Food composition table
FFQ: Food frequency questionnaire
FH-CVD: Family history of cardiovascular disease
FPG: Fasting plasma glucose
HEI: Healthy eating index
LOCF: Last observation carried forward
MAQ: Metabolic activity equivalent
MICE: Multivariate imputations by chained equations
PAL: Physical activity level
PCA: Principal component analysis
PH: Proportional hazard
RCT: Randomized clinical trials
SBP: Systolic blood pressure
TLGS: Tehran lipid and glucose study
USDA: United state department of agriculture
