# Supplementary material for: The association of priori and posteriori dietary patterns with the risk of incident hypertension: Tehran Lipid and Glucose Study
Source: J Transl Med. 2021 Jan 25;19:44. doi: 10.1186/s12967-021-02704-w (PMC7836444; doi:10.1186/s12967-021-02704-w)
Supplement: Supplementary file 1 — Additional file 1: Table S1. Baseline characteristics of participants who developed and did not develop hypertension in the participants of the Tehran Lipid and Glucose Study (n=4793). Table S2. Baseline characteristics of male participants by quartiles of different dietary patterns in the Tehran Lipid and Glucose Study (n=1986). Table S3. Baseline characteristics of female participants by quartiles of different dietary patterns in the Tehran Lipid and Glucose Study (n=2807). Table S4. Baseline dietary intake of participants who developed and did not develop hypertension in the Tehran Lipid and Glucose Study (n=4793). Table S5. Dietary intakes of participants by quartiles of extracted dietary patterns by PCA among male participants of the Tehran Lipid and Glucose Study (n=1986). Table S6. Dietary intakes of participants by quartiles of a priori dietary patterns among male participants of the Tehran Lipid and Glucose Study (n=1986). Table S7. Dietary intakes of participants by quartiles of extracted dietary patterns by PCA among female participants of the Tehran Lipid and Glucose Study (n=2807). Table S8. Dietary intakes of participants by quartiles of a priori dietary patterns among female participants of the Tehran Lipid and Glucose Study (n=2807).. [file 12967_2021_2704_MOESM1_ESM.docx]

| **Additional file 1: Table S1.** Baseline characteristics of participants who developed and did not develop hypertension in the participants of the Tehran Lipid and Glucose Study (n=4793) | | | | | | | | | |
| --- | --- | --- | --- | --- | --- | --- | --- | --- | --- |
|  | **Men** | | | **Women** | | | **Total population** | | |
|  | **With hypertension**  **n=343** | **Without hypertension**  **n=1643** | **P value** | **With hypertension**  **n=384** | **Without hypertension**  **n=2423** | **P value** | **With hypertension**  **n=727** | **Without hypertension**  **n=4066** | **P value** |
| Age (years) | 46.8 (13.6) | 38.9 (13.1) | <0.001 | 48.9 (11.1) | 36.2 (11.4) | <0.001 | 47.9 (12.4) | 37.3 (12.2) | <0.001 |
| BMI (kg/m^2^) | 27.9 (4.1) | 26.0 (3.9) | <0.001 | 30.4 (4.9) | 26.4 (4.6) | <0.001 | 29.3 (4.7) | 26.2 (4.3) | <0.001 |
| TG (mmol/L) | 1.9 (1.2) | 1.7 (1.3) | <0.001 | 1.7 (0.9) | 1.3 (0.7) | <0.001 | 1.8(1.1) | 1.4 (1.1) | <0.001 |
| SBP (mmHg) | 120.6 (9.9) | 110.8 (10.2) | <0.001 | 117.5 (11.4) | 103.9 (10.7) | <0.001 | 118.9 (10.8) | 106.7 (11.1) | <0.001 |
| DBP (mmHg) | 80.5 (6.1) | 74.5 (7.7) | <0.001 | 77.2 (7.1) | 70.1 (8.3) | <0.001 | 78.7 (6.8) | 71.9 (8.3) | <0.001 |
| PAL (MET-minutes per week) | 704.2 (1016.6) | 676.1 (1045.0) | 0.643 | 520.7 (727.5) | 464.6 (700.7) | 0.159 | 607.3 (880.1) | 550.1 (862.8) | 0.106 |
| FH-CVD (Yes %) | 18 (5.2) | 58 (3.5) | 0.162 | 23 (6.0) | 153 (6.3) | 0.910 | 41 (5.6) | 211 (5.2) | 0.589 |
| Diabetes (Yes %) | 36 (10.5) | 86 (5.2) | <0.001 | 49 (12.8) | 88 (3.6) | <0.001 | 85 (11.7) | 174 (4.3) | <0.001 |
| Current smoker % | 109 (31.8) | 677 (41.2) | 0.001 | 24 (6.2) | 259 (10.7) | 0.006 | 133 (18.3) | 936 (23.0) | 0.005 |
| Values are presented as mean (SD) for continuous variables, and frequency (%) for categorical variables.  **BMI**: body mass index; **DBP**: diastolic blood pressure; **FH-CVD**: family history of CVD; **MET**: metabolic equivalent task; **PAL**: physical activity level; **SBP**: systolic blood pressure; **SD**: standard deviation; **TG**: triglycerides | | | | | | | | | |

| **Additional file 1: Table S2. Baseline characteristics of male participants by quartiles of different dietary patterns in the Tehran Lipid and Glucose Study (n=1986)** | | | | | | | | | | | |
| --- | --- | --- | --- | --- | --- | --- | --- | --- | --- | --- | --- |
|  | **Quartile of healthy dietary pattern score** | | | |  |  | **Quartiles of unhealthy dietary pattern score** | | | |  |
|  | **1** | **2** | **3** | **4** | **P trend** |  | **1** | **2** | **3** | **4** | **P trend** |
| Age (years) | 38.0 (12.8) | 39.6 (13.4) | 42.2 (13.6) | 42.8 (14.1) | <0.001 |  | 47.5 (13.8) | 43.0 (13) | 39.4 (13) | 35.5 (12) | <0.001 |
| BMI (kg/m^2^) | 25.9 (4.1) | 26.4 (4.1) | 26.4 (4.0) | 26.9 (3.8) | <0.001 |  | 26.3 (4) | 26.4 (3.9) | 26.5 (4.1) | 26.3 (4.1) | 0.810 |
| TG (mmol/L) | 1.7 (1.1) | 1.7 (1.9) | 1.7 (1.0) | 1.7 (1.0) | 0.821 |  | 1.7 (1.0) | 1.9 (2.0) | 1.7 (1.0) | 1.7 (1.1) | 0.168 |
| SBP (mmHg) | 111.8 (10.4) | 112.5 (11.1) | 113.4 (10.9) | 112.9 (10.6) | 0.033 |  | 113.4 (11.6) | 112.6 (10.6) | 112.4 (10.6) | 112.3 (10.6) | 0.133 |
| DBP (mmHg) | 75.0 (8.2) | 75.4 (7.8) | 76.3 (7.6) | 75.4 (7.3) | 0.135 |  | 75.2 (7.8) | 75.7 (7.9) | 75.7 (7.8) | 75.4 (7.7) | 0.920 |
| PAL (MET-minutes per week) | 569 (905) | 653(1034) | 729 (1061) | 846(1196) | <0.001 |  | 691(971) | 620(897) | 677(967) | 719 (1205) | 0.371 |
| FH-CVD (Yes %) | 27 (4.4) | 19 (3.5) | 12 (2.7) | 18 (4.7) | 0.374 |  | 13 (3.7) | 16 (3.6) | 20 (3.9) | 27 (4.0) | 0.723 |
| Diabetes (Yes %) | 30 (4.9) | 35 (6.4) | 26 (5.8) | 31 (8.2) | 0.063 |  | 48 (13.7) | 33 (7.4) | 22 (4.2) | 19 (2.8) | <0.001 |
| Current smoker % | 254 (41.2) | 234 (42.9) | 175 (39.3) | 123 (32.5) | 0.007 |  | 89 (25.4) | 147 (32.9) | 223 (43.1) | 327 (48.8) | <0.001 |
|  | **Quartile of DASH score** | | | |  |  | **Quartile of HEI-2015 score** | | | |  |
|  | **1** | **2** | **3** | **4** | **P trend** |  | **1** | **2** | **3** | **4** | **P trend** |
| Age (years) | 35 (11.9) | 38.8 (12.1) | 42.2 (13.5) | 46.8 (13.9) | <0.001 |  | 37.4 (12.8) | 39.0 (13.0) | 41.6 (13.5) | 44.3 (14.2) | <0.001 |
| BMI (kg/m^2^) | 26.3 (4.4) | 26.3 (3.9) | 26.2 (3.8) | 26.6 (3.8) | 0.243 |  | 26.1 (4.3) | 26.2 (3.9) | 26.3 (4) | 26.9 (3.8) | 0.007 |
| TG (mmol/L) | 1.7 (1.0) | 1.7 (1.0) | 1.8 (2.0) | 1.7 (1.0) | 0.296 |  | 1.6 (0.9) | 1.7 (1.9) | 1.7 (1.0) | 1.8 (1.3) | <0.001 |
| SBP (mmHg) | 111.8 (10.3) | 111.8 (10.9) | 112.9 (10.8) | 114 (11.1) | <0.001 |  | 111.8 (10.6) | 112.4 (10.8) | 112.8 (10.7) | 113.6 (11) | 0.008 |
| DBP (mmHg) | 75.1 (7.8) | 75.5 (7.8) | 75.6 (8) | 75.9 (7.5) | 0.109 |  | 75.2 (8.1) | 75.3 (7.9) | 75.7 (7.4) | 76 (7.7) | 0.078 |
| PAL (MET-minutes per week) | 645(1174) | 579(884) | 715(1044) | 789(970) | 0.010 |  | 644(1051) | 619(1050) | 723(1074) | 761(966) | 0.035 |
| FH-CVD (Yes %) | 27(4.5) | 15 (3.5) | 17 (3.3) | 17 (3.8) | 0.519 |  | 27 (4.9) | 20 (3.7) | 18 (3.8) | 11 (2.7) | 0.093 |
| Diabetes (Yes %) | 20 (3.3) | 24 (5.6) | 41(8.0) | 37(8.4) | <0.001 |  | 25 (4.6) | 33 (6.0) | 35 (7.3) | 29 (7.0) | 0.065 |
| Current smoker % | 290 (48.0) | 186 (43.4) | 191 (37.4) | 119 (26.9) | <0.001 |  | 242 (44.1) | 214 (39.1) | 189 (39.5) | 141 (34.2) | 0.004 |
| **BMI**: body mass index; **DASH**: Dietary Approaches to Stop Hypertension; **DBP**: diastolic blood pressure; **FH-CVD**: family history of CVD; **HEI-2015**: healthy eating index; **MET**: metabolic equivalent task; **PAL**: physical activity level; **SBP**: systolic blood pressure; **SD**: standard deviation; **TG**: triglycerides; **PCA-1**: principal component analysis-factor 1; **PCA-2**: principal component analysis-factor 2.  Values are reported as mean(SD) unless otherwise presented. **P trends** were calculated by treating quartiles of dietary patterns scores as a continuous variable, and including this variable in the logistic and linear regression model for categorical and continuous variables, respectively. | | | | | | | | | | | |

| **Additional file 1: Table S3. Baseline characteristics of female participants by quartiles of different dietary patterns in the Tehran Lipid and Glucose Study (n=2807)** | | | | | | | | | | | |
| --- | --- | --- | --- | --- | --- | --- | --- | --- | --- | --- | --- |
|  | **Quartiles of healthy dietary pattern score** | | | |  |  | **Quartiles of unhealthy dietary pattern score** | | | |  |
|  | **1** | **2** | **3** | **4** | **P trend** |  | **1** | **2** | **3** | **4** | **P trend** |
| Age (years) | 35.4 (12.1) | 37.2 (12.1) | 38.4 (12.3) | 40 (11.9) | <0.001 |  | 42.6 (12.1) | 38.1 (12.0) | 36.1 (11.4) | 32.8 (10.7) | <0.001 |
| BMI (kg/m^2^) | 26.3 (5.0) | 26.7 (4.8) | 27.1 (4.9) | 27.6 (4.8) | <0.001 |  | 28.0 (4.9) | 27.0 (4.8) | 26.6 (4.8) | 25.8 (4.8) | <0.001 |
| TG (mmol/L) | 1.2 (0.7) | 1.3 (0.7) | 1.4 (0.9) | 1.4 (0.8) | <0.001 |  | 1.5 (0.8) | 1.3 (0.8) | 1.3 (0.8) | 1.2 (0.8) | <0.001 |
| SBP (mmHg) | 104.1 (11.8) | 105.5 (11.8) | 106.5 (12) | 106.5 (11.7) | <0.001 |  | 106.9 (12.4) | 106.7 (11.8) | 105.1 (11.7) | 103.5 (10.7) | <0.001 |
| DBP (mmHg) | 69.9 (8.9) | 70.9 (8.4) | 71.6 (8.3) | 71.8 (8.5) | <0.001 |  | 71.5 (8.4) | 71.7 (8.5) | 70.8 (8.8) | 70.1 (8.2) | 0.001 |
| PAL (MET-minutes per week) | 383.0  (613.2) | 435.4  (665.4) | 426.8  (671.7) | 607.1  (800.8) | <0.001 |  | 510.8  (680.0) | 500.0  (763.2) | 442.1  (613.6) | 410.1 (759.9) | 0.004 |
| FH-CVD (Yes %) | 36 (6.2) | 42 (6.4) | 39 (5.2) | 59 (7.2) | 0.598 |  | 59 (7.0) | 36 (4.8) | 39 (5.7) | 42 (8.0) | 0.560 |
| Diabetes (Yes %) | 24 (4.1) | 30 (4.6) | 34 (4.5) | 49 (6.0) | 0.121 |  | 65 (7.7) | 40 (5.3) | 19 (2.8) | 13 (2.5) | <0.001 |
| Current smoker % | 88 (15.1) | 67 (10.3) | 71 (9.4) | 57 (7.0) | <0.001 |  | 54 (6.4) | 68 (9.1) | 71 (10.4) | 90 (17.0) | <0.001 |
|  | **Quartile of DASH score** | | | |  |  | **Quartile of HEI-2015 score** | | | |  |
|  | **1** | **2** | **3** | **4** | **P trend** |  | **1** | **2** | **3** | **4** | **P trend** |
| Age (years) | 33.3 (11) | 36.4 (11.8) | 38.0 (11.7) | 42.6 (12.2) | <0.001 |  | 36.5 (11.6) | 36.7 (11.9) | 37.8 (12.2) | 40.6 (12.5) | <0.001 |
| BMI (kg/m^2^) | 25.9 (4.9) | 26.5 (5) | 27.1 (4.8) | 28.0 (4.8) | <0.001 |  | 26.6 (4.9) | 26.7 (5) | 26.8 (4.8) | 27.7 (4.8) | <0.001 |
| TG (mmol/L) | 1.2 (0.7) | 1.3 (0.8) | 1.3 (0.8) | 1.4 (0.9) | <0.001 |  | 1.3 (0.7) | 1.3 (0.9) | 1.3 (0.8) | 1.4 (0.8) | 0.032 |
| SBP (mmHg) | 103 (11.4) | 105.7 (11.5) | 106.6 (12.1) | 107.1 (11.8) | <0.001 |  | 104.6 (11.6) | 105.1 (12) | 106 (11.9) | 107.1 (11.8) | <0.001 |
| DBP (mmHg) | 70.1 (8.6) | 70.8 (8.7) | 71.6 (8.5) | 71.8 (8.3) | <0.001 |  | 71.2 (8.6) | 70.6 (8.4) | 70.9 (8.8) | 71.9 (8.3) | 0.074 |
| PAL (MET-minutes per week) | 374.7 (571.0) | 431.7 (571.0) | 447.9 (571.0) | 596.3 (571.0) | <0.001 |  | 355.9 (552.1) | 428.5 (626.4) | 500.2 (757.4) | 588.2 (812.9) | <0.001 |
| FH-CVD (Yes %) | 48 (7.4) | 32 (6.4) | 49 (6.0) | 47 (5.6) | <0.001 |  | 48 (7.2) | 45 (6.6) | 40 (5.7) | 43 (5.7) | 0.180 |
| Diabetes (Yes %) | 21 (3.2) | 23 (4.6) | 31 (3.8) | 62 (7.4) | 0.001 |  | 20 (3.0) | 26 (3.8) | 41 (5.8) | 50 (6.6) | <0.001 |
| Current smoker % | 117 (18.0) | 54 (10.7) | 65 (8.0) | 47 (5.6) | <0.001 |  | 80 (12.1) | 82 (12.0) | 73 (10.4) | 48 (6.4) | <0.001 |
| **BMI**: body mass index; **DASH**: Dietary Approaches to Stop Hypertension; **DBP**: diastolic blood pressure; **FH-CVD**: family history of CVD; **HEI-2015**: healthy eating index; **MET**: metabolic equivalent task; **PAL**: physical activity level; **SBP**: systolic blood pressure; **SD**: standard deviation; **TG:** triglycerides; **PCA-1**: principal component analysis-factor 1; **PCA-2**: principal component analysis-factor 2  Values are reported as mean (SD) unless otherwise presented. **P trends** were calculated by treating quartiles of dietary patterns scores as a continuous variable, and including this variable in the logistic and linear regression model for categorical and continuous variables, respectively. | | | | | | | | | | | |

| **Additional file 1: Table S4. Baseline dietary intake of participants who developed and did not develop hypertension in the Tehran Lipid and Glucose Study (n=4793)** | | | | | | | | | |
| --- | --- | --- | --- | --- | --- | --- | --- | --- | --- |
|  | **Men** | | | **Women** | | | **Total population** | | |
|  | **With hypertension**  **n=343** | **Without hypertension**  **n=1643** | **P value** | **With hypertension**  **n=384** | **Without hypertension**  **n=2423** | **P value** | **With hypertension**  **n=727** | **Without hypertension**  **n=4066** | **P value** |
| Liquid oils (gr/1000 kcal) **participants of the Tehran Lipid and Glucose Study (n=4793)** | 3.7 (4.20) | 3.7 (2.6) | 0.880 | 4.5 (3.7) | 4.3 (3.1) ) | 0.373 | 4.1 (2.9) | 4.2 (3.9) | 0.660 |
| Solid oils (gr/1000 kcal) | 5.1 (5.3) | 5.7 (5.3) | 0.064 | 5.7 (6.2) | 6.0 (6.30 | 0.386 | 5.9 (5.9) | 5.4 (5.8) | 0.055 |
| Added sugars (% of energy) | 5.1 (3.3) | 5.7 (3.4) | 0.001 | 4.2 (2.8) | 4.6 (2.8) | 0.009 | 5.1 (3.1) | 4.6 (3.1) | <0.001 |
| Legumes* | 0.2 (0.2) | 0.2 (0.1) | 0.351 | 0.2 (0.2) | 0.1 (0.1) | <0.001 | 0.1 (0.1) | 0.2 (0.2) | 0.001 |
| Red meats* | 0.3 (0.2) | 0.4 (0.2) | 0.001 | 0.3 (0.2) | 0.4 (0.3) | 0.042 | 0.4 (0.3) | 0.3 (0.2) | <0.001 |
| Nuts and seeds* | 0.2 (0.2) | 0.2 (0.3) | 0.313 | 0.2 (0.4) | 0.2 (0.3) | 0.028 | 0.2 (0.3) | 0.2 (0.3) | 0.164 |
| Fast foods* | 0.2 (0.1) | 0.2 (0.2) | 0.045 | 0.1 (0.1) | 0.2 (0.2) | <0.001 | 0.1 (0.1) | 0.2 (0.2) | <0.001 |
| Low fat dairy products* | 0.6 (0.4) | 0.5 (0.3) | 0.009 | 0.7 (0.4) | 0.6 (0.4) | <0.001 | 0.7 (0.4) | 0.6 (0.4) | <0.001 |
| High fat dairy products* | 0.3 (0.3) | 0.3 (0.2) | 0.331 | 0.3 (0.3) | 0.4(0.3) | 0.001 | 0.3 (0.3) | 0.3 (0.3) | 0.001 |
| Green vegetables* | 0.3 (0.2) | 0.2 (0.2) | 0.025 | 0.3 (0.2) | 0.2 (0.2) | 0.002 | 0.3 (0.2) | 0.3 (0.1) | 0.001 |
| Yellow and red vegetables* | 0.3 (0.2) | 0.3 (0.2) | 0.079 | 0.4 (0.3) | 0.3 (0.3) | <0.001 | 0.3 (0.1) | 0.3 (0.2) | 0.001 |
| Other vegetables* | 0.6 (0.4) | 0.5 (0.3) | 0.001 | 0.9 (0.5) | 0.8 (0.4) | <0.001 | 0.8 (0.5) | 0.7 (0.4) | <0.001 |
| Refined grains* | 0.6 (0.4) | 0.5 (0.3) | 0.555 | 3.7 (1.7) | 3.7 (1.6) | 0.962 | 4.0 (1.8) | 4.0 (1.6) | 0.780 |
| Whole grains* | 1.0 (1.2) | 0.9 (1.1) | 0.030 | 0.8 (0.9) | 0.7 (0.9) | 0.085 | 0.9 (1.1) | 0.8 (0.9) | 0.003 |
| Salty snacks* | 0.1 (0.1) | 0.1 (0.3) | 0.313 | 0.1 (0.2) | 0.1 (0.2) | 0.476 | 0.1 (0.1) | 0.1 (0.2) | 0.169 |
| Poultry and fish* | 0.6 (0.5) | 0.6 (0.5) | 0.257 | 0.6 (0.5) | 0.6 (0.5) | 0.512 | 0.6 (0.6) | 0.6 (0.5) | 0.202 |
| Tea and coffee* | 701 (515) | 643 (488) | 0.046 | 578 (437) | 515 (413) | 0.006 | 636 (479) | 566 (449) | <0.001 |
| Carbonated drinks* | 54.5 (76.6) | 64.3 (82.1) | 0.035 | 29.1 (46.8) | 37.4 (55.8) | 0.002 | 41.1 (63.9) | 48.2 (68.9) | 0.006 |
| Total fruit* | 1.1 (0.7) | 1.0 (0.7) | 0.218 | 1.3 (0.8) | 1.3 (0.8) | 0.919 | 1.2 (0.8) | 1.2 (0.8) | 0.695 |
| Fruits juice* | 0.1 (0.2) | 0.1 (0.2) | 0.798 | 0.1 (0.2) | 0.1 (0.2) | 0.376 | 0.1 (0.2) | 0.1 (0.2) | 0.439 |
| Salt (gr/1000 kcal) | 2.2 (2.1) | 2.3 (2.3) | 0.602 | 3.5 (12.8) | 2.8 (2.1) | 0.290 | 2.9 (9.4) | 2.6 (2.1) | 0.395 |
| Total energy (kcal/day) | 2451.8 (730.2) | 2511.9 (718.1) | 0.160 | 2260.5 (682.8) | 2335.6 (705.4) | 0.051 | 2350.8 (711.5) | 2406.8 (715.7) | 0.051 |
| Values are presented as mean (SD) for continuous variables, * Servings per 1000 Kcal | | | | | | | | | |

| **Additional file 1: Table S5. Dietary intakes of participants by quartiles of extracted dietary patterns by PCA among male participants of the Tehran Lipid and Glucose Study (n=1986)** | | | | | | | | | | | | | | | | | | | | | | | | | | | | | | | | | | |
| --- | --- | --- | --- | --- | --- | --- | --- | --- | --- | --- | --- | --- | --- | --- | --- | --- | --- | --- | --- | --- | --- | --- | --- | --- | --- | --- | --- | --- | --- | --- | --- | --- | --- | --- |
|  | | **Quartile of healthy dietary pattern score** | | | | | | | | | | | | | |  | | | **Quartile of unhealthy dietary pattern score** | | | | | | | | | | | | |  | | |
|  | | **1** | | | **2** | | | | **3** | | | | **4** | | | **P trend** | | | **1** | | | | **2** | | | **3** | | | **4** | | | **P trend** | | |
| Liquid oils(gr/1000 kcal) | | 3.4 (2.6) | | | 3.6 (2.6) | | | | 3.9 (2.8) | | | | 4.2 (4.0) | | | <0.001 | | | 3.7 (2.9) | | | | 3.4 (2.4) | | | 3.7 (2.7) | | | 3.9 (3.6) | | | 0.104 | | |
| Solid oils (gr/1000 kcal) | | 6.8 (6.3) | | | 5.5 (4.8) | | | | 5.2 (4.8) | | | | 4.3 (4.5) | | | <0.001 | | | 3.8 (5.2) | | | | 5.0 (5.1) | | | 6.0 (5.3) | | | 6.6 (5.3) | | | <0.001 | | |
| Added sugars (% of energy) | | 6.6 (4.0) | | | 5.7 (3.4) | | | | 5.3 (3.0) | | | | 4.6 (2.5) | | | <0.001 | | | 3.2 (2.0) | | | | 4.6 (2.7) | | | 5.7 (3.0) | | | 7.7 (3.6) | | | <0.001 | | |
| Legumes* | | 0.1 (0.1) | | | 0.2 (0.1) | | | | 0.2 (0.2) | | | | 0.2 (0.2) | | | <0.001 | | | 0.2 (0.2) | | | | 0.2 (0.2) | | | 0.2 (0.1) | | | 0.2 (0.1) | | | <0.001 | | |
| Red meats* | | 0.4 (0.2) | | | 0.4 (0.3) | | | | 0.4 (0.3) | | | | 0.4 (0.3) | | | 0.483 | | | 0.3 (0.2) | | | | 0.4 (0.3) | | | 0.4 (0.2) | | | 0.4 (0.3) | | | <0.001 | | |
| Nuts and seeds* | | 0.2 (0.2) | | | 0.2 (0.2) | | | | 0.2 (0.3) | | | | 0.3 (0.3) | | | <0.001 | | | 0.2 (0.2) | | | | 0.2 (0.2) | | | 0.2 (0.2) | | | 0.2 (0.3) | | | <0.001 | | |
| Fast foods* | | 0.2 (0.2) | | | 0.2 (0.2) | | | | 0.2 (0.1) | | | | 0.1 (0.1) | | | <0.001 | | | 0.1 (0.1) | | | | 0.1 (0.1) | | | 0.2 (0.1) | | | 0.3 (0.2) | | | <0.001 | | |
| Low fat dairy products* | | 0.5 (0.3) | | | 0.6 (0.3) | | | | 0.6 (0.3) | | | | 0.6 (0.3) | | | <0.001 | | | 0.7 (0.4) | | | | 0.6 (0.4) | | | 0.5 (0.3) | | | 0.5 (0.3) | | | <0.001 | | |
| High fat dairy products* | | 0.3 (0.3) | | | 0.3 (0.3) | | | | 0.3 (0.2) | | | | 0.3 (0.2) | | | 0.151 | | | 0.2 (0.2) | | | | 0.3 (0.3) | | | 0.3 (0.3) | | | 0.4 (0.3) | | | <0.001 | | |
| Green vegetables* | | 0.2 (0.1) | | | 0.2 (0.2) | | | | 0.3 (0.2) | | | | 0.4 (0.2) | | | <0.001 | | | 0.3 (0.2) | | | | 0.3 (0.2) | | | 0.2 (0.2) | | | 0.2 (0.1) | | | <0.001 | | |
| Yellow and red vegetables* | | 0.2 (0.1) | | | 0.2 (0.1) | | | | 0.3 (0.2) | | | | 0.4 (0.2) | | | <0.001 | | | 0.4 (0.3) | | | | 0.3 (0.2) | | | 0.2 (0.2) | | | 0.2 (0.1) | | | <0.001 | | |
| Other vegetables* | | 0.4 (0.2) | | | 0.5 (0.3) | | | | 0.7 (0.3) | | | | 0.8 (0.4) | | | <0.001 | | | 0.7 (0.4) | | | | 0.6 (0.4) | | | 0.5 (0.3) | | | 0.5 (0.3) | | | <0.001 | | |
| Refined grains* | | 5.4 (1.8) | | | 4.6 (1.5) | | | | 4.1 (1.4) | | | | 3.3 (1.3) | | | <0.001 | | | 4.5 (1.9) | | | | 4.9 (1.8) | | | 4.6 (1.7) | | | 4.2 (1.5) | | | <0.001 | | |
| Whole grains* | | 0.7 (0.9) | | | 0.9 (1.2) | | | | 1.0 (1.1) | | | | 1.0 (1.1) | | | <0.001 | | | 1.5 (1.6) | | | | 0.9 (1.1) | | | 0.9 (0.9) | | | 0.6 (0.7) | | | <0.001 | | |
| Salty snacks* | | 0.1 (0.1) | | | 0.1 (0.4) | | | | 0.1 (0.1) | | | | 0.1 (0.1) | | | 0.393 | | | 0.1 (0.1) | | | | 0.1 (0.5) | | | 0.1 (0.1) | | | 0.2 (0.1) | | | <0.001 | | |
| Poultry and fish* | | 0.5 (0.4) | | | 0.6 (0.4) | | | | 0.6 (0.5) | | | | 0.8 (0.9) | | | <0.001 | | | 0.6 (0.5) | | | | 0.6 (0.6) | | | 0.6 (0.5) | | | 0.6 (0.6) | | | 0. 276 | | |
| Tea and coffee* | | 577.5(456.5) | | | 644.4(489.8) | | | | 697.2(504.3) | | | | 735.1(525.2) | | | <0.001 | | | 490.2(359.2) | | | | 598.3(410.3) | | | 635.4(436.9) | | | 787.8(600.4) | | | <0.001 | | |
| Carbonated drinks* | | 74.3 (98.6) | | | 59.8 (73.1) | | | | 62 (78.5) | | | | 48.5 (58.8) | | | <0.001 | | | 10.7 (11.7) | | | | 22.8 (20.6) | | | 45.3 (40.5) | | | 129.9(103.5) | | | <0.001 | | |
| Total fruit* | | 0.7 (0.5) | | | 1.0 (0.7) | | | | 1.2 (0.7) | | | | 1.5 (0.9) | | | <0.001 | | | 1.1 (0.8) | | | | 1.0 (0.7) | | | 1.1 (0.8) | | | 1.0 (0.7) | | | 0.340 | | |
| Fruits juice* | | 0.08 (0.11) | | | 0.11 (0.13) | | | | 0.13 (0.16) | | | | 0.18 (0.24) | | | <0.001 | | | 0.07 (0.09) | | | | 0.10 (0.13) | | | 0.12 (0.16) | | | 0.15 (0.20) | | | <0.001 | | |
| Salt (gr/1000 kcal) | | 2.1 (1.9) | | | 2.3 (2.0) | | | | 2.5 (1.8) | | | | 2.4 (1.9) | | | 0.001 | | | 1.9 (1.5) | | | | 2.1 (1.8) | | | 2.2 (1.9) | | | 2.7 (2.2) | | | <0.001 | | |
| Total energy (kcal/day) | | 2072.3(629.6) | | | 2426.8 (621) | | | | 2706.9(627.5) | | | | 3065.8 (620) | | | <0.001 | | | 1841.2(520.5) | | | | 2178.6(557.2) | | | 2560.4(596.5) | | | 3017.4(590.3) | | | <0.001 | | |
| **P trends** were calculated by treating quartiles of dietary patterns scores as a continuous variable, and including this variable in the logistic and linear regression model for categorical and continuous variables, respectively. * Servings per 1000 Kcal | | | | | | | | | | | | | | | | | | | | | | | | | | | | | | | | | | |
| **Additional file 1: Table S6. Dietary intakes of participants by quartiles of a priori dietary patterns among male participants of the Tehran Lipid and Glucose Study (n=1986)** | | | | | | | | | | | | | | | | | | | | | | | | | | | | | | | | |  |  |
|  | | **DASH score quartile** | | | | | | | | | | | | | | | | | **HEI-2015 score quartile** | | | | | | | | | | | | | |  |  |
|  | | **1** | | | **2** | | **3** | | | **4** | | | | **P trend** | |  | | | **1** | | **2** | | | **3** | | | **4** | | | **P trend** | | |  |  |
| Liquid oils (gr/1000 kcal) | | 3.7 (2.8) | | | 3.7 (2.5) | | 3.8 (3.7) | | | 3.6 (2.6) | | | | 0.546 | |  | | | 3.4 (2.5) | | 3.7 (2.7) | | | 3.9 (2.7) | | | 3.9 (4.0) | | | 0.001 | | |  |  |
| Solid oils (gr/1000 kcal) | | 6.6 (5.7) | | | 6.1 (5.3) | | 5.7 (5.3) | | | 3.7 (4.3) | | | | <0.001 | |  | | | 7.0 (6.1) | | 5.5 (4.8) | | | 5.1 (4.9) | | | 4.6 (4.9) | | | <0.001 | | |  |  |
| Added sugars (% of energy) | | 6.8 (3.8) | | | 5.8 (3.3) | | 5.3 (3.3) | | | 4.4 (2.8) | | | | <0.001 | |  | | | 6.7 (4) | | 5.8 (3.5) | | | 5.1 (2.8) | | | 4.8 (2.8) | | | <0.001 | | |  |  |
| Legumes* | | 0.2 (0.1) | | | 0.2 (0.1) | | 0.2 (0.1) | | | 0.2 (0.2) | | | | <0.001 | |  | | | 0.1 (0.1) | | 0.2 (0.2) | | | 0.2 (0.1) | | | 0.2 (0.2) | | | <0.001 | | |  |  |
| Red meats* | | 0.5 (0.3) | | | 0.4 (0.2) | | 0.3 (0.2) | | | 0.3 (0.3) | | | | <0.001 | |  | | | 0.4 (0.2) | | 0.4 (0.3) | | | 0.4 (0.2) | | | 0.4 (0.3) | | | 0.534 | | |  |  |
| Nuts and seeds* | | 0.2 (0.2) | | | 0.2 (0.3) | | 0.2 (0.2) | | | 0.3 (0.3) | | | | <0.001 | |  | | | 0.2 (0.2) | | 0.2 (0.3) | | | 0.2 (0.3) | | | 0.2 (0.3) | | | <0.001 | | |  |  |
| Fast foods* | | 0.3 (0.2) | | | 0.2 (0.1) | | 0.1 (0.2) | | | 0.1 (0.1) | | | | <0.001 | |  | | | 0.2 (0.2) | | 0.2 (0.2) | | | 0.2 (0.2) | | | 0.1 (0.2) | | | <0.001 | | |  |  |
| Low fat dairy products* | | 0.4 (0.3) | | | 0.5 (0.3) | | 0.6 (0.3) | | | 0.7 (0.3) | | | | <0.001 | |  | | | 0.5 (0.4) | | 0.5 (0.3) | | | 0.6 (0.3) | | | 0.6 (0.3) | | | <0.001 | | |  |  |
| High fat dairy products* | | 0.3 (0.3) | | | 0.3 (0.3) | | 0.3 (0.2) | | | 0.3 (0.3) | | | | 0.010 | |  | | | 0.3 (0.3) | | 0.3 (0.3) | | | 0.3 (0.2) | | | 0.3 (0.2) | | | <0.001 | | |  |  |
| Green vegetables* | | 0.2 (0.1) | | | 0.2 (0.1) | | 0.3 (0.2) | | | 0.3 (0.2) | | | | <0.001 | |  | | | 0.2 (0.1) | | 0.2 (0.2) | | | 0.3 (0.2) | | | 0.3 (0.2) | | | <0.001 | | |  |  |
| Yellow and red vegetables* | | 0.2 (0.1) | | | 0.2 (0.2) | | 0.3 (0.2) | | | 0.4 (0.2) | | | | <0.001 | |  | | | 0.2 (0.1) | | 0.3 (0.2) | | | 0.3 (0.2) | | | 0.3 (0.2) | | | <0.001 | | |  |  |
| Other vegetables* | | 0.4 (0.3) | | | 0.5 (0.3) | | 0.6 (0.3) | | | 0.8 (0.4) | | | | <0.001 | |  | | | 0.4 (0.3) | | 0.6 (0.3) | | | 0.6 (0.3) | | | 0.7 (0.4) | | | <0.001 | | |  |  |
| Refined grains* | | 5.1 (1.7) | | | 4.6 (1.7) | | 4.3 (1.6) | | | 3.7 (1.4) | | | | <0.001 | |  | | | 5.4 (1.6) | | 4.9 (1.5) | | | 4.2 (1.4) | | | 3.0 (1.2) | | | <0.001 | | |  |  |
| Whole grains* | | 0.5 (0.8) | | | 0.9 (1.2) | | 1.0 (1.2) | | | 1.3 (1.1) | | | | <0.001 | |  | | | 0.4 (0.6) | | 0.6 (0.7) | | | 1.2 (1.2) | | | 1.7 (1.4) | | | <0.001 | | |  |  |
| Salty snacks* | | 0.1 (0.1) | | | 0.1 (0.1) | | 0.1 (0.4) | | | 0.1 (0.1) | | | | <0.001 | |  | | | 0.1 (0.4) | | 0.1 (0.1) | | | 0.1 (0.1) | | | 0.1 (0.1) | | | 0.001 | | |  |  |
| Poultry and fish* | | 0.6 (0.6) | | | 0.6 (0.5) | | 0.6 (0.5) | | | 0.6 (0.6) | | | | 0.381 | |  | | | 0.5 (0.4) | | 0.6 (0.4) | | | 0.6 (0.6) | | | 0.8 (0.8) | | | <0.001 | | |  |  |
| Tea and coffee* | | 628.4(492.8) | | | 666.8(536.8) | | 675.6(489.7) | | | 646.1(452.4) | | | | 0.414 | |  | | | 654.8(520.8) | | 674.3(510.2) | | | 627.4(448.1) | | | 651.0(483.2) | | | 0.541 | | |  |  |
| Carbonated drinks* | | 104.9(105.1) | | | 62.6(66.4) | | 47.8(64.8) | | | 22.1(33.5) | | | | <0.001 | |  | | | 81.3(101.1) | | 63.2(81.4) | | | 52.8(65.1) | | | 48.5(61.3) | | | <0.001 | | |  |  |
| Total fruit* | | 0.7 (0.5) | | | 1.0 (0.7) | | 1.1 (0.7) | | | 1.6 (0.9) | | | | <0.001 | |  | | | 0.7 (0.5) | | 1.0 (0.7) | | | 1.2 (0.7) | | | 1.4 (0.9) | | | <0.001 | | |  |  |
| Fruits juice* | | 0.09 (0.12) | | | 0.12 (0.17) | | 0.12 (0.17) | | | 0.14 (0.19) | | | | <0.001 | |  | | | 0.09 (0.12) | | 0.10 0.15) | | | 0.13 (0.17) | | | 0.16 (0.20) | | | <0.001 | | |  |  |
| Salt (gr/1000 kcal) | | 2.8 (2.2) | | | 2.3 (1.8) | | 2.2 (1.8) | | | 1.7 (1.6) | | | | <0.001 | |  | | | 2.9 (2.2) | | 2.3 (1.9) | | | 2.1 (1.7) | | | 1.7 (1.5) | | | <0.001 | | |  |  |
| Total energy(kcal/day) | | 2515(714.7) | | | 2519 (741.0) | | 2519 (726.0) | | | 2446 (700.8) | | | | 0.184 | |  | | | 2449 (698.7) | | 2470 (741.0) | | | 2568(721.4) | | | 2536(714.8) | | | 0.012 | | |  |  |
| **DASH**: Dietary Approaches to Stop Hypertension; **HEI-2015**: healthy eating index  **P trends** were calculated by treating quartiles of dietary patterns scores as a continuous variable, and including this variable in the logistic and linear regression model for categorical and continuous variables, respectively. * Servings per 1000 Kcal | | | | | | | | | | | | | | | | | | | | | | | | | | | | | | | | |  |  |
| **Additional file 1: Table S7. Dietary intakes of participants by quartiles of extracted dietary patterns by PCA among female participants of the Tehran Lipid and Glucose Study (n=2807)** | | | | | | | | | | | | | | | | | | | | | | | | | | | | | | | | |  |  |
|  | | | | **Quartile of healthy dietary pattern score** | | | | | | | | | |  | | | | **Quartile of unhealthy dietary pattern score** | | | | | | | | | | | |  | | |  |  |
|  | | | | **1** | | | **2** | | | **3** | | **4** | | **P trend** | | | | **1** | | | **2** | | | **3** | | | **4** | | | **P trend** | | |  |  |
| Liquid oils (gr/1000 kcal) | | | | 3.8 (2.9) | | | 4.3 (3) | | | 4.3 (2.9) | | 4.8 (3.8) | | <0.001 | | | | 4.5 (3) | | | 4.3 (3.1) | | | 4.2 (3.1) | | | 4.6 (3.9) | | | 0.893 | | |  |  |
| Solid oils (gr/1000 kcal) | | | | 7.9 (8.1) | | | 6.1 (6) | | | 5.6 (5.7) | | 5 (5.3) | | <0.001 | | | | 4.0 (5.2) | | | 6.2 (6.4) | | | 7.0 (6.2) | | | 7.7 (7.2) | | | <0.001 | | |  |  |
| Added sugars (% of energy) | | | | 5.3 (3.4) | | | 4.9 (2.8) | | | 4.6 (2.6) | | 3.8 (2.3) | | <0.001 | | | | 3.1 (2) | | | 4.4 (2.5) | | | 5.0 (2.5) | | | 6.8 (3.3) | | | <0.001 | | |  |  |
| Legumes* | | | | 0.2 (0.1) | | | 0.2 (0.1) | | | 0.2 (0.2) | | 0.2 (0.2) | | <0.001 | | | | 0.2 (0.2) | | | 0.2 (0.2) | | | 0.2 (0.2) | | | 0.2 (0.1) | | | <0.001 | | |  |  |
| Red meats* | | | | 0.4 (0.3) | | | 0.4 (0.3) | | | 0.4 (0.3) | | 0.4 (0.2) | | <0.001 | | | | 0.3 (0.2) | | | 0.4 (0.2) | | | 0.4 (0.3) | | | 0.4 (0.3) | | | <0.001 | | |  |  |
| Nuts and seeds* | | | | 0.1 (0.2) | | | 0.2 (0.3) | | | 0.2 (0.2) | | 0.3 (0.4) | | <0.001 | | | | 0.2 (0.2) | | | 0.2 (0.3) | | | 0.2 (0.3) | | | 0.3 (0.4) | | | <0.001 | | |  |  |
| Fast foods* | | | | 0.2 (0.2) | | | 0.2 (0.2) | | | 0.2 (0.2) | | 0.1 (0.1) | | <0.001 | | | | 0.1 (0.1) | | | 0.1 (0.1) | | | 0.2 (0.2) | | | 0.3 (0.2) | | | <0.001 | | |  |  |
| Low fat dairy products* | | | | 0.6 (0.4) | | | 0.6 (0.4) | | | 0.7 (0.4) | | 0.7 (0.4) | | <0.001 | | | | 0.8 (0.5) | | | 0.6 (0.3) | | | 0.6 (0.3) | | | 0.5 (0.3) | | | <0.001 | | |  |  |
| High fat dairy products* | | | | 0.4 (0.3) | | | 0.4 (0.3) | | | 0.3 (0.3) | | 0.3 (0.3) | | 0.001 | | | | 0.3 (0.3) | | | 0.4 (0.3) | | | 0.4 (0.3) | | | 0.4 (0.3) | | | <0.001 | | |  |  |
| Green vegetables* | | | | 0.2 (0.1) | | | 0.2 (0.1) | | | 0.3 (0.2) | | 0.4 (0.2) | | <0.001 | | | | 0.4 (0.2) | | | 0.3 (0.2) | | | 0.3 (0.2) | | | 0.2 (0.2) | | | <0.001 | | |  |  |
| Yellow and red vegetables* | | | | 0.2 (0.1) | | | 0.3 (0.2) | | | 0.3 (0.2) | | 0.5 (0.3) | | <0.001 | | | | 0.5 (0.3) | | | 0.3 (0.2) | | | 0.3 (0.2) | | | 0.2 (0.2) | | | <0.001 | | |  |  |
| Other vegetables* | | | | 0.5 (0.3) | | | 0.7 (0.3) | | | 0.8 (0.4) | | 1.0 (0.5) | | <0.001 | | | | 1.0 (0.5) | | | 0.8 (0.4) | | | 0.7 (0.3) | | | 0.6 (0.3) | | | <0.001 | | |  |  |
| Refined grains* | | | | 4.8 (1.8) | | | 3.9 (1.4) | | | 3.6 (1.4) | | 2.8 (1.3) | | <0.001 | | | | 3.7 (1.7) | | | 3.9 (1.7) | | | 3.7 (1.6) | | | 3.4 (1.4) | | | <0.001 | | |  |  |
| Whole grains* | | | | 0.6 (0.9) | | | 0.7 (0.8) | | | 0.7 (1) | | 0.8 (1) | | <0.001 | | | | 1.0 (1.2) | | | 0.7 (0.9) | | | 0.6 (0.7) | | | 0.4 (0.5) | | | <0.001 | | |  |  |
| Salty snacks* | | | | 0.1 (0.2) | | | 0.1 (0.1) | | | 0.1 (0.1) | | 0.2 (0.3) | | 0.008 | | | | 0.1 (0.1) | | | 0.1 (0.1) | | | 0.2 (0.4) | | | 0.2 (0.2) | | | <0.001 | | |  |  |
| Poultry and fish* | | | | 0.5 (0.4) | | | 0.6 (0.4) | | | 0.6 (0.5) | | 0.7 (0.6) | | <0.001 | | | | 0.6 (0.5) | | | 0.6 (0.5) | | | 0.6 (0.5) | | | 0.6 (0.6) | | | 0.359 | | |  |  |
| Tea and coffee* | | | | 456 (381.4) | | | 507(397.1) | | | 538(416) | | 572 (450) | | <0.001 | | | | 436 (309) | | | 511 (402) | | | 566 (441) | | | 627 (516) | | | <0.001 | | |  |  |
| Carbonated drinks* | | | | 41.1(64) | | | 36.2(55.3) | | | 38.2(56) | | 31.1(44.9) | | 0.002 | | | | 9.4(11.5) | | | 19.2(18.9) | | | 37.4(36.5) | | | 102(87.2) | | | <0.001 | | |  |  |
| Total fruit* | | | | 0.8 (0.6) | | | 1.1 (0.7) | | | 1.3 (0.9) | | 1.7 (1) | | <0.001 | | | | 1.3 (0.9) | | | 1.2 (0.8) | | | 1.3 (0.9) | | | 1.2 (0.9) | | | 0.021 | | |  |  |
| Fruits juice* | | | | 0.06 (0.08) | | | 0.10 (0.17) | | | 0.11 (0.22) | | 0.16 (0.22) | | <0.001 | | | | 0.08 (0.12) | | | 0.10 (0.14) | | | 0.12 (0.20) | | | 0.16 (0.24) | | | <0.001 | | |  |  |
| Salt (gr/1000 kcal) | | | | 3.1 (10.5) | | | 2.8 (2.2) | | | 3.0 (2) | | 3.1 (2.2) | | 0.747 | | | | 2.7 (8.7) | | | 2.9 (2.1) | | | 3.1 (2.1) | | | 3.4 (2.5) | | | 0.011 | | |  |  |
| Total energy(kcal/day) | | | | 1828 (564.5) | | | 2073(562) | | | 2397(588) | | 2814(654) | | <0.001 | | | | 1848(562) | | | 2191(548) | | | 2588(600) | | | 2944(604) | | | <0.001 | | |  |  |
| **P trends** were calculated by treating quartiles of dietary patterns scores as a continuous variable, and including this variable in the logistic and linear regression model for categorical and continuous variables, respectively. * Servings per 1000 Kcal | | | | | | | | | | | | | | | | | | | | | | | | | | | | | | | | |  |  |

| **Additional file 1: Table S8: Dietary intakes of participants by quartiles of a priori dietary patterns among female participants of the Tehran Lipid and Glucose Study (n=2807)** | | | | | | | | | | | |
| --- | --- | --- | --- | --- | --- | --- | --- | --- | --- | --- | --- |
|  | **DASH score quartile** | | | |  |  | **HEI-2015 score quartile** | | | |  |
| WOMEN | **1** | **2** | **3** | **4** | **P trend** |  | **1** | **2** | **3** | **4** | **P trend** |
| Liquid oils (gr/1000 kcal) | 4.4 (3.2) | 4.3 (3.1) | 4.3 (3.3) | 4.5 (3.2) | 0.663 |  | 4.0 (3) | 4.2 (3.3) | 4.4 (2.9) | 4.9 (3.6) | <0.001 |
| Solid oils (gr/1000 kcal) | 7.5 (7) | 6.7 (6.8) | 5.8 (6) | 4.6 (5.5) | <0.001 |  | 7.6 (7.6) | 6.2 (6.1) | 5.6 (5.6) | 4.8 (5.5) | <0.001 |
| Added sugars (% of energy) | 5.5 (3) | 4.8 (2.8) | 4.5 (2.8) | 3.8 (2.5) | <0.001 |  | 5.2 (3.4) | 4.7 (2.8) | 4.6 (2.6) | 4.0 (2.3) | <0.001 |
| Legumes* | 0.1 (0.1) | 0.2 (0.1) | 0.2 (0.1) | 0.3 (0.2) | <0.001 |  | 0.1 (0.1) | 0.2 (0.1) | 0.2 (0.2) | 0.3 (0.2) | <0.001 |
| Red meats* | 0.5 (0.3) | 0.4 (0.3) | 0.4 (0.3) | 0.3 (0.2) | <0.001 |  | 0.4 (0.3) | 0.4 (0.3) | 0.4 (0.3) | 0.4 (0.3) | 0.353 |
| Nuts and seeds* | 0.2 (0.3) | 0.2 (0.2) | 0.2 (0.3) | 0.3 (0.3) | <0.001 |  | 0.2 (0.2) | 0.2 (0.3) | 0.2 (0.2) | 0.3 (0.3) | <0.001 |
| Fast foods* | 0.2 (0.2) | 0.2 (0.2) | 0.1 (0.1) | 0.1 (0.1) | <0.001 |  | 0.2 (0.2) | 0.2 (0.2) | 0.2 (0.2) | 0.1 (0.1) | <0.001 |
| Low fat dairy products* | 0.5 (0.3) | 0.6 (0.3) | 0.7 (0.4) | 0.8 (0.4) | <0.001 |  | 0.6 (0.4) | 0.6 (0.4) | 0.6 (0.4) | 0.7 (0.4) | <0.001 |
| High fat dairy products* | 0.4 (0.3) | 0.3 (0.3) | 0.3 (0.3) | 0.3 (0.3) | <0.001 |  | 0.4 (0.3) | 0.3 (0.3) | 0.3 (0.3) | 0.3 (0.3) | <0.001 |
| Green vegetables* | 0.2 (0.2) | 0.3 (0.2) | 0.3 (0.2) | 0.4 (0.2) | <0.001 |  | 0.2 (0.2) | 0.3 (0.2) | 0.3 (0.2) | 0.4 (0.2) | <0.001 |
| Yellow and red vegetables* | 0.2 (0.2) | 0.3 (0.2) | 0.4 (0.3) | 0.5 (0.3) | <0.001 |  | 0.3 (0.2) | 0.3 (0.2) | 0.4 (0.3) | 0.4 (0.3) | <0.001 |
| Other vegetables* | 0.6 (0.3) | 0.7 (0.4) | 0.8 (0.4) | 1.0 (0.5) | <0.001 |  | 0.7 (0.4) | 0.8 (0.4) | 0.8 (0.4) | 0.9 (0.5) | <0.001 |
| Refined grains* | 4.4 (1.7) | 4.2 (1.7) | 3.7 (1.5) | 2.9 (1.4) | <0.001 |  | 4.9 (1.6) | 4.2 (1.5) | 3.5 (1.3) | 2.4 (1) | <0.001 |
| Whole grains* | 0.4 (0.6) | 0.6 (0.9) | 0.8 (1.1) | 1.0 (1) | <0.001 |  | 0.3 (0.4) | 0.5 (0.7) | 0.7 (0.9) | 1.3 (1.2) | <0.001 |
| Salty snacks* | 0.2 (0.2) | 0.1 (0.1) | 0.1 (0.1) | 0.1 (0.3) | <0.001 |  | 0.1 (0.2) | 0.1 (0.2) | 0.1 (0.1) | 0.1 (0.4) | 0.151 |
| Poultry and fish* | 0.6 (0.5) | 0.6 (0.6) | 0.6 (0.5) | 0.6 (0.5) | 0.602 |  | 0.5 (0.4) | 0.5 (0.4) | 0.7 (0.6) | 0.7 (0.6) | <0.001 |
| Tea and coffee* | 513 (420) | 513 (404) | 522(408) | 539(431) | 0.189 |  | 503(404) | 542 (430) | 527(409) | 522(423) | 0.570 |
| Carbonated drinks* | 67.7(70.5) | 39.7(56.1) | 29.8(47) | 16.1(30.5) | <0.001 |  | 41.1(60.7) | 39.3(57.3) | 36.2(52.4) | 29.3(48) | <0.001 |
| Total fruit* | 0.8 (0.7) | 1.0 (0.8) | 1.3 (0.9) | 1.7 (0.9) | <0.001 |  | 0.8 (0.6) | 1.1 (0.8) | 1.4 (0.8) | 1.7 (1) | <0.001 |
| Fruits juice* | 0.09 (0.15) | 0.10 (0.16) | 0.11 (0.17) | 0.13 (0.20) | <0.001 |  | 0.08 (0.16) | 0.09 (0.14) | 0.12 (0.17) | 0.14 (0.21) | <0.001 |
| Salt (gr/1000 kcal) | 4.0 (10) | 3.1 (2.4) | 2.8 (1.9) | 2.4 (1.7) | <0.001 |  | 3.9 (9.9) | 3.1 (2.2) | 2.7 (1.9) | 2.3 (1.7) | <0.001 |
| Total energy(kcal/day) | 2316(689) | 2254(711) | 2326(696) | 2375(713) | 0.032 |  | 2204(664) | 2313(722) | 2333(696) | 2436(707) | <0.001 |
| **DASH**: Dietary Approaches to Stop Hypertension; **HEI-2015**: healthy eating index  **P trends** were calculated by treating quartiles of dietary patterns scores as a continuous variable, and including this variable in the logistic and linear regression model for categorical and continuous variables, respectively. * Servings per 1000 Kcal | | | | | | | | | | | |
